# Supplementary material for: H3K4 methylation at active genes mitigates transcription-replication conflicts during replication stress
Source: Nat Commun. 2020 Feb 10;11:809. doi: 10.1038/s41467-020-14595-4 (PMC7010754; doi:10.1038/s41467-020-14595-4)
Supplement: Supplementary file 1 — Supplementary Information [file 41467_2020_14595_MOESM1_ESM.pdf]

Supplementary Information

H3K4 methylation at active genes mitigates transcription-replication conflicts during replication stress

By Chong *et al.*

Supplementary Figure 1

a

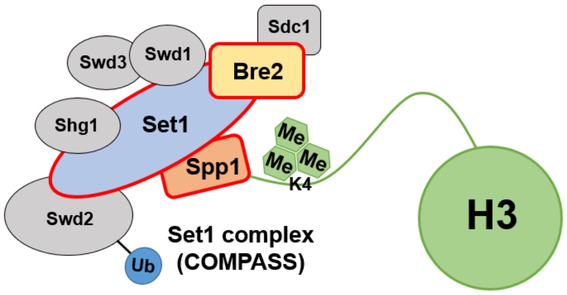

b

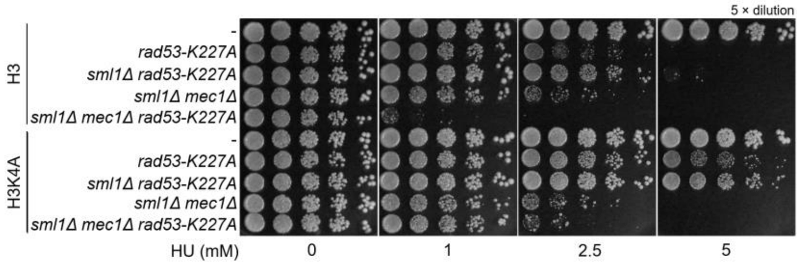

c

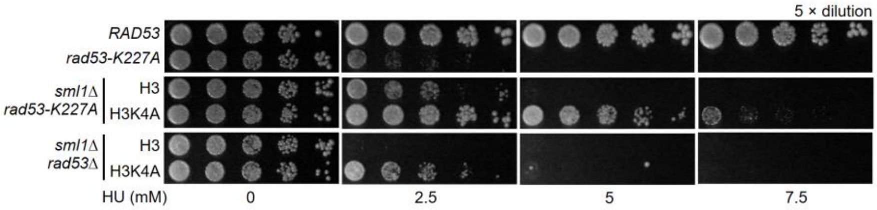

d

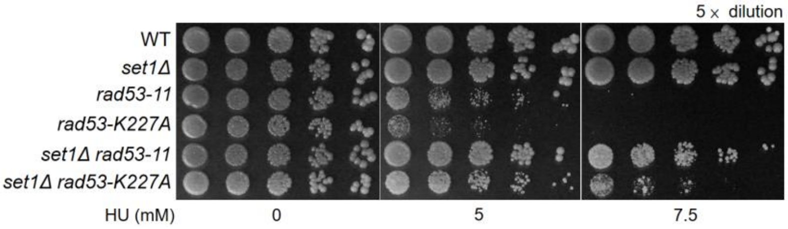

Supplementary Figure 1. *rad53* HU sensitivity is negatively correlated with H3K4 methylation levels and independent of Mec1 signaling.

a Schematic representation of the Set1 Complex. HU sensitivity was determined with **b** WT (*MEC1 RAD53*), *sml1Δ mec1Δ*, *rad53-K227A* and *sml1Δ mec1Δ rad53-K227A* backgrounds; **c** *sml1Δ rad53Δ and sml1Δ rad53-K227A* with either H3 or H3K4A mutation; **d** *set1Δ* and kinase-dead *rad53* mutant with mutation of K227A or G653E (*rad53-11*).

Supplementary Figure 2

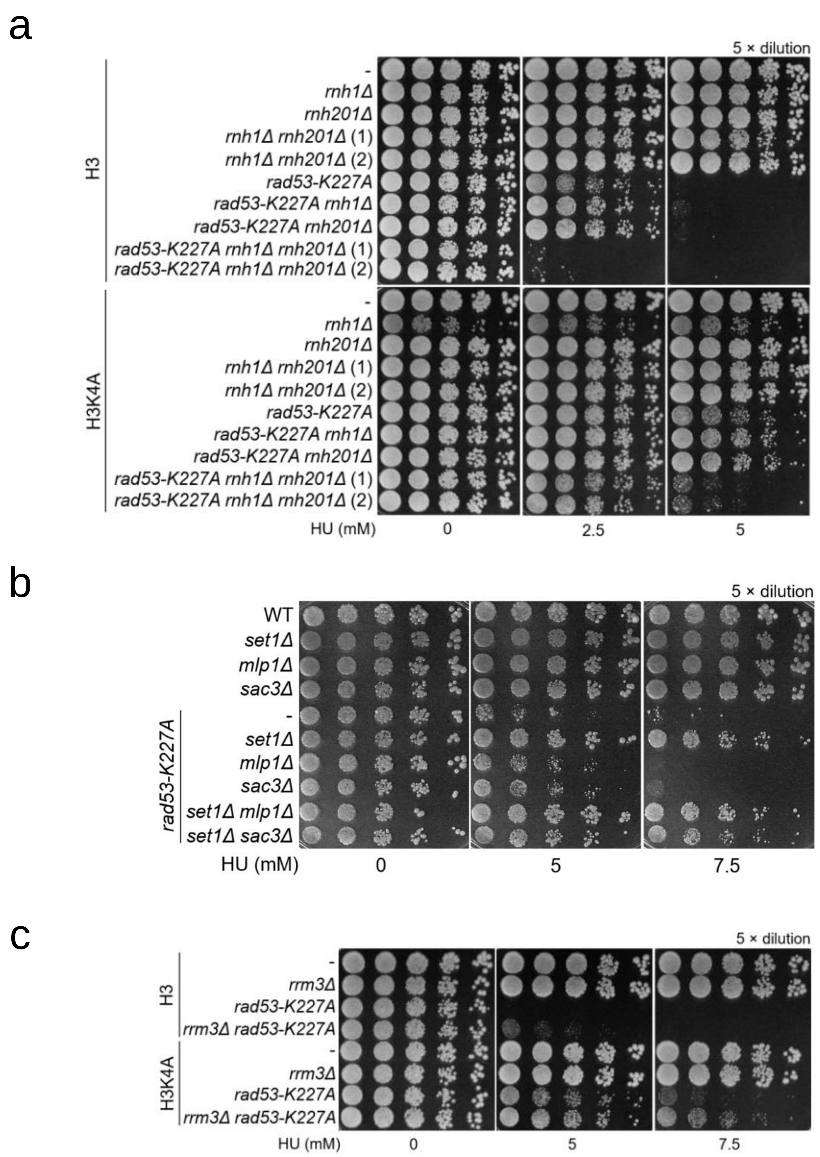

Supplementary Figure 2. The interaction between ablation of H3K4 methylation and *rad53* mutation is independent of the R-loop and known *rad53* HU-sensitivity suppressors.

a-c HU sensitivity was determined with **a**, RNase H mutation(s); **b** gene gating gene mutation and **c** Rad53-suppressed helicase mutation in *RAD53* and *rad53-K227A* backgrounds with normal (H3) or ablated (H3K4A or with *set1Δ*) H3K4 methylation.

Supplementary Figure 3

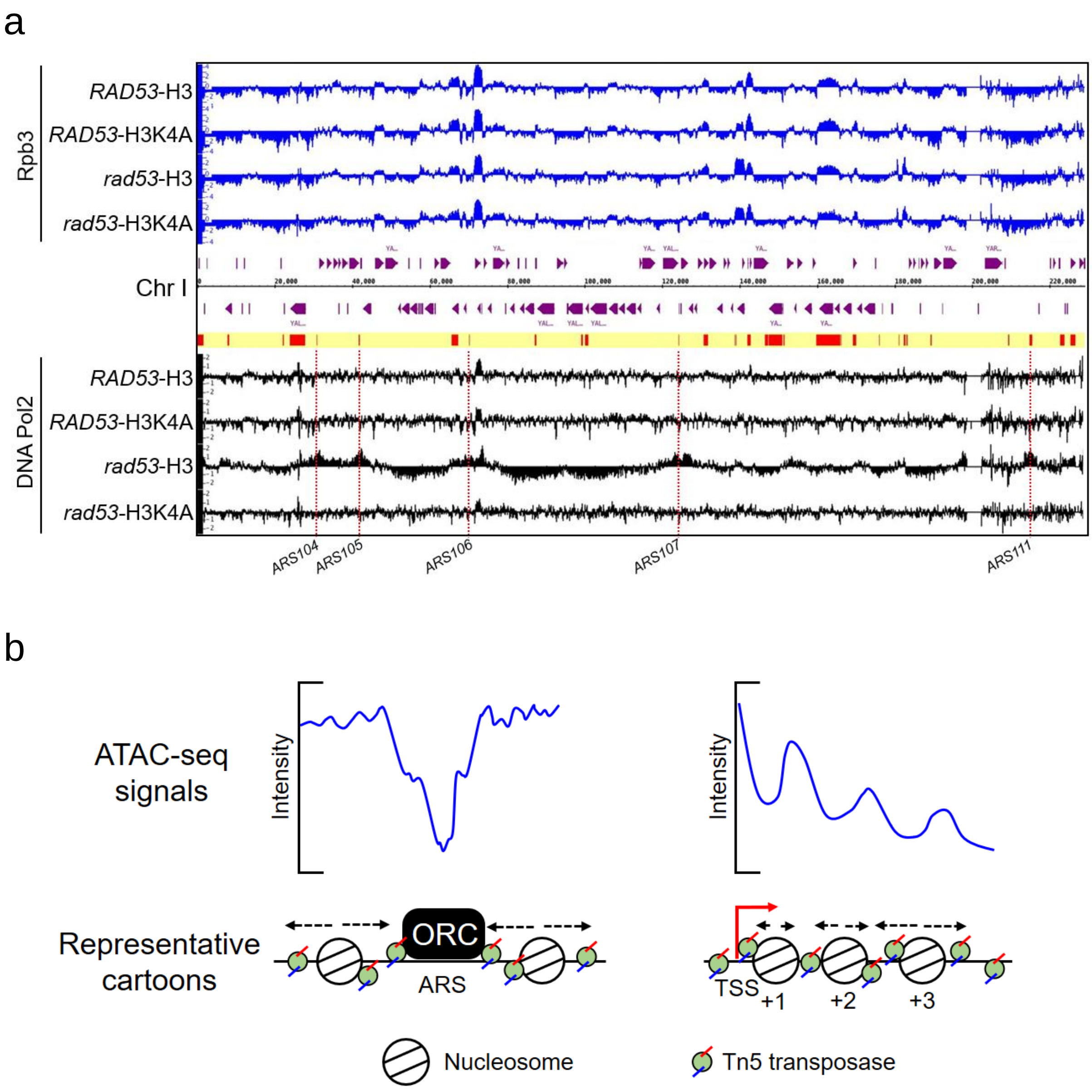

Supplementary Figure 3. The restored-replication progression of *rad53* mutants in HU by losing H3K4me is unconcerned with alterations in chromatin structure.

a Rbp3 and DNA Pol2 ChIP-seq tracks of *RAD53*-H3, *RAD53*-H3K4A, *rad53*-H3 and *rad53*-H3K4A strains on chromosome I (Chr I). *rad53* refers to *rad53-K227A* mutant. Red dashed lines indicate ARSs with stalled DNA Pol2 in *rad53* cells. **b** Representative cartoons to illustrate ATAC-seq signal found on ORC-bound origins and TSS regions; with longer arrows on nucleosomes representing nucleosomes that are less consistently positioned.

Supplementary Figure 4

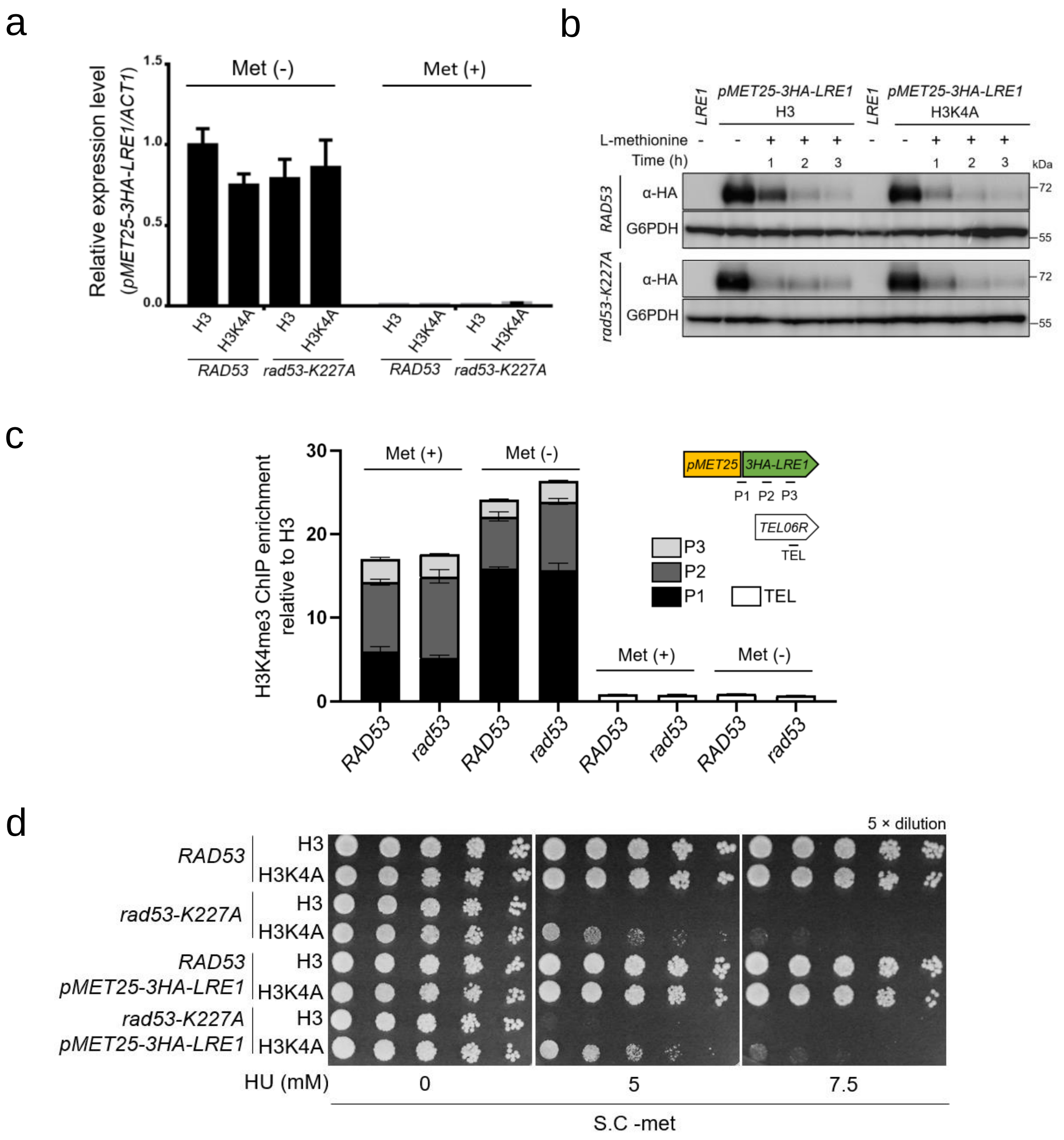

Supplementary Figure 4. Evaluation of the *pMET25-3HA-LRE1-ARS305* TRC system.

a The gene expression levels of *pMET25-3HA-LRE1* of *RAD53* and *rad53-K227A* cells with either normal H3 or H3K4A mutation after incubation in S.C.-met medium (Met (-), activation) or S.C. medium (Met (+), suppression) **b** Protein levels resulting from activated and suppressed *pMET25-3HA-LRE1* gene in strains similar to **a**, cells were initially cultured in S.C.-met medium with addition of L-methionine (0.005%, w/v) into medium for the indicated time. **c** H3K4me3 deposition on *pMET25-3HA-LRE1* on indication regions under suppressed (met+) and activated (met-) conditions **d** Induction of additional HO-oriented TRCs between *pMET25-3HA-LRE1* transcription and replication fork from *ARS305* do not affect cell survival upon HU treatment. Error bars represent ± SEM (n = 3 technical replicates).

Supplementary Table 1: Strains used in this study

Strains	Number	Genotype	Reference/source
MSY421 (wild-type)	CSY0016	*MATa ura3-52 leu2-3,112 trp1 his3 Δ[HHT1-HHF1] Δ[HHT2-HHF2]* pMS329-*copy I [URA3, HHT1-HHF1]*	David Allis's lab[1]
RAD53-H3	CSY0019	*MATa ura3-52 leu2-3,112 trp1 his3 Δ[HHT1-HHF1] Δ[HHT2-HHF2]* pMS337 *[CEN4 ARS1 LEU2 HHT1-HHF1]*	This study (Fig. 1a, S1b-c, 7a, S2a)
RAD53-H3K4A	CSY0020	*MATa ura3-52 leu2-3,112 trp1 his3 Δ[HHT1-HHF1] Δ[HHT2-HHF2]* pMS337 *[CEN4 ARS1 LEU2 hht1-H3K4A-HHF1]*	This study (Fig. 1a, 7a, S1b, S2a)
RAD53-H3R2A	CSY0024	*MATa ura3-52 leu2-3,112 trp1 his3 Δ[HHT1-HHF1] Δ[HHT2-HHF2]* pMS337 *[CEN4 ARS1 LEU2 hht1-H3R2A-HHF1]*	This study (Fig. 1a, 7a)
RAD53-H3K36A	CSY0022	*MATa ura3-52 leu2-3,112 trp1 his3 Δ[HHT1-HHF1] Δ[HHT2-HHF2]* pMS337 *[CEN4 ARS1 LEU2 hht1-H3K36A-HHF1]*	This study (Fig. 1a, 7a)
RAD53-H3K79A	CSY0023	*MATa ura3-52 leu2-3,112 trp1 his3 Δ[HHT1-HHF1] Δ[HHT2-HHF2]* pMS337 *[CEN4 ARS1 LEU2 hht1-H3K79A-HHF1]*	This study (Fig. 1a, 7a)
rad53-K227A-H3	CSY0025	*MATa ura3-52 leu2-3,112 trp1 his3 Δ[HHT1-HHF1] Δ[HHT2-HHF2] natNT2-rad53-K227A pMS337 [CEN4 ARS1 LEU2 HHT1-HHF1]*	This study (Fig. 1a, S1b-c, S2A)
rad53-K227A-H3K4A	CSY0026	*MATa ura3-52 leu2-3,112 trp1 his3 Δ[HHT1-HHF1] Δ[HHT2-HHF2] natNT2-rad53-K227A pMS337 [CEN4 ARS1 LEU2 HHT1-H3K4A-HHF1]*	This study (Fig. 1a, S1b, S2a)
rad53-K227A-H3R2A	CSY0030	*MATa ura3-52 leu2-3,112 trp1 his3 Δ[HHT1-HHF1] Δ[HHT2-HHF2] natNT2-rad53-K227A pMS337 [CEN4 ARS1 LEU2 HHT1-H3R2A-HHF1]*	This study (Fig. 1a)
rad53-K227A-H3K79A	CSY0028	*MATa ura3-52 leu2-3,112 trp1 his3 Δ[HHT1-HHF1] Δ[HHT2-HHF2] natNT2-rad53-K227A pMS337 [CEN4 ARS1 LEU2 HHT1-H3K36A-HHF1]*	This study (Fig. 1a)
rad53-K227A-H3K79A	CSY0029	*MATa ura3-52 leu2-3,112 trp1 his3 Δ[HHT1-HHF1] Δ[HHT2-HHF2] natNT2-rad53-K227A pMS337 [CEN4 ARS1 LEU2 HHT1-H3K79A-HHF1]*	This study (Fig. 1a)
H3	CSY0252	*MATa ura3-52 leu2-3,112 trp1 his3 Δ[HHT1-HHF1] Δ[HHT2-HHF2] bar1::HIS3 natNT2-pMET25-3HA-LRE1* pMS337 *[CEN4 ARS1 LEU2 HHT1-HHF1]*	This study (Fig. 1b-d, 4e, 5b-c, 6b-c, 7c-d, 7f, S2c, S4a-d)
rad53-K227A-H3	CSY0186	*MATa ura3-52 leu2-3,112 trp1 his3 Δ[HHT1-HHF1] Δ[HHT2-HHF2] kanMX4-rad53-K227A bar1::HIS3 natNT2-pMET25-3HA-LRE1* pMS337 *[CEN4 ARS1 LEU2 HHT1-HHF1]*	This study (Fig. 1b-e, 3a, 4h, 5b-c, S2c, S4a, S4c-d)
rad53-K227A set1Δ-H3	CSY0302	*MATa ura3-52 leu2-3,112 trp1 his3 Δ[HHT1-HHF1] Δ[HHT2-HHF2] kanMX4-rad53-K227A bar1::HIS3 natNT2-pMET25-3HA-LRE1 set1::HPH* pMS337 *[CEN4 ARS1 LEU2 HHT1-HHF1]*	This study (Fig. 1b-e, 3a, 4h)
rad53-K227A bre2Δ-H3	CSY0303	*MATa ura3-52 leu2-3,112 trp1 his3 Δ[HHT1-HHF1] Δ[HHT2-HHF2] kanMX4-rad53-K227A bar1::HIS3 natNT2-pMET25-3HA-LRE1 bre2::HPH* pMS337 *[CEN4 ARS1 LEU2 HHT1-HHF1]*	This study (Fig. 1b-e, 3a, 4h)
rad53-K227A spp1Δ-H3	CSY0304	*MATa ura3-52 leu2-3,112 trp1 his3 Δ[HHT1-HHF1] Δ[HHT2-HHF2] kanMX4-rad53-K227A bar1::HIS3 natNT2-pMET25-3HA-LRE1 spp1::HPH* pMS337 *[CEN4 ARS1 LEU2 HHT1-HHF1]*	This study (Fig. 1b-e, 3a, 4h)
set1Δ-H3	CSY0299	*MATa ura3-52 leu2-3,112 trp1 his3 Δ[HHT1-HHF1] Δ[HHT2-HHF2] bar1::HIS3 natNT2-pMET25-3HA-LRE1 set1::HPH* pMS337 *[CEN4 ARS1 LEU2 HHT1-HHF1]*	This study (Fig. 1c, 5c, 7a)
bre2Δ-H3	CSY0300	*MATa ura3-52 leu2-3,112 trp1 his3 Δ[HHT1-HHF1] Δ[HHT2-HHF2] bar1::HIS3 natNT2-pMET25-3HA-LRE1 bre2::HPH* pMS337 *[CEN4 ARS1 LEU2 HHT1-HHF1]*	This study (Fig. 1c, 7a)

spp1Δ-H3	CSY0301	*MATa ura3-52 leu2-3,112 trp1 his3 Δ[HHT1-HHF1] Δ[HHT2-HHF2] bar1::HIS3 natNT2-pMET25-3HA-LRE1 spp1::HPH* pMS337 *[CEN4 ARS1 LEU2 HHT1-HHF1]*	This study (Fig. 1c, 7a)
H3K4A	CSY0253	*MATa ura3-52 leu2-3,112 trp1 his3 Δ[HHT1-HHF1] Δ[HHT2-HHF2] bar1::HIS3 natNT2-pMET25-3HA-LRE1* pMS337 *[CEN4 ARS1 LEU2 HHT1-HHF1]*	This study (Fig. 1d, 4e, 5b, 6b-c, 7c-d, 7f, S2c, S4a-b, S4d)
rad53-K227A-H3K4A	CSY0187	*MATa ura3-52 leu2-3,112 trp1 his3 Δ[HHT1-HHF1] Δ[HHT2-HHF2] kanMX4-rad53-K227A bar1::HIS3 natNT2-pMET25-3HA-LRE1* pMS337 *[CEN4 ARS1 LEU2 hht1-H3K4A-HHF1]*	This study (Fig. 1d, 3a, 4f, 5b, 6b, S2c, S4a, S4c-d)
RAD53-H3 (DNA Pol2-3×FLAG)	CSY0489	*MATa ura3-52 leu2-3,112 trp1 his3 Δ[HHT1-HHF1] Δ[HHT2-HHF2] POL2-3×FLAG-kanMX4* pMS337 *[CEN4 ARS1 LEU2 HHT1-HHF1]*	This study (Fig. 2, 3b, S3a)
RAD53-H3K4A (DNA Pol2-3×FLAG)	CSY0490	*MATa ura3-52 leu2-3,112 trp1 his3 Δ[HHT1-HHF1] Δ[HHT2-HHF2] POL2-3×FLAG-kanMX4* pMS337 *[CEN4 ARS1 LEU2 hht1-H3K4A-HHF1]*	This study (Fig. 2, 3b, S3a)
rad53-K227A-H3 (DNA Pol2-3×FLAG)	CSY0491	*MATa ura3-52 leu2-3,112 trp1 his3 Δ[HHT1-HHF1] Δ[HHT2-HHF2] natNT2-rad53-K227A POL2-3×FLAG-kanMX4 pMS337 [CEN4 ARS1 LEU2 HHT1-HHF1]*	This study (Fig. 2, 3b, S3a)
rad53-K227A-H3K4A (DNA Pol2-3×FLAG)	CSY0492	*MATa ura3-52 leu2-3,112 trp1 his3 Δ[HHT1-HHF1] Δ[HHT2-HHF2] natNT2-rad53-K227A POL2-3×FLAG-kanMX4 pMS337 [CEN4 ARS1 LEU2 HHT1-H3K4A-HHF1]*	This study (Fig. 2, 3b, S3a)
RAD53-H3	CSY0095	*MATa ura3-52 leu2-3,112 trp1 his3 Δ[HHT1-HHF1] Δ[HHT2-HHF2] bar1::HIS3* pMS337 *[CEN4 ARS1 LEU2 HHT1-HHF1]*	This study (Fig. 3c-f, 4c, S4d)
RAD53-H3K4A	CSY0096	*MATa ura3-52 leu2-3,112 trp1 his3 Δ[HHT1-HHF1] Δ[HHT2-HHF2] bar1::HIS3* pMS337 *[CEN4 ARS1 LEU2 hht1-H3K4A-HHF1]*	This study (Fig. 3c-f, 4c, S4d)
rad53-K227A-H3	CSY0101	*MATa ura3-52 leu2-3,112 trp1 his3 Δ[HHT1-HHF1] Δ[HHT2-HHF2] natNT2-rad53-K227A bar1::HIS3 pMS337 [CEN4 ARS1 LEU2 HHT1-HHF1]*	This study (Fig. 3c-f, 4c, S4d)
rad53-K227A-H3K4A	CSY0102	*MATa ura3-52 leu2-3,112 trp1 his3 Δ[HHT1-HHF1] Δ[HHT2-HHF2] natNT2-rad53-K227A bar1::HIS3 pMS337 [CEN4 ARS1 LEU2 HHT1-H3K4A-HHF1]*	This study (Fig. 3c-f, 4c, S4d)
rad53-K227A HO-pMET25-3HA-LRE1-H3	CSY0457	*MATa ura3-52 leu2-3,112 trp1 his3 Δ[HHT1-HHF1] Δ[HHT2-HHF2] natNT2-rad53-K227A bar1::HIS3 HO-pMET25-3HA-LRE1* pMS337 *[CEN4 ARS1 LEU2 HHT1-HHF1]*	This study (Fig. 4g)
rad53-K227A HO-pMET25-3HA-LRE1-H3K4A	CSY0458	*MATa ura3-52 leu2-3,112 trp1 his3 Δ[HHT1-HHF1] Δ[HHT2-HHF2] natNT2-rad53-K227A bar1::HIS3 HO-pMET25-3HA-LRE1* pMS337 *[CEN4 ARS1 LEU2 hht1-H3K4A-HHF1]*	This study (Fig. 4g)
rad53-K227A CD-pMET25-3HA-LRE1-H3	CSY0459	*MATa ura3-52 leu2-3,112 trp1 his3 Δ[HHT1-HHF1] Δ[HHT2-HHF2] natNT2-rad53-K227A bar1::HIS3 CD-pMET25-3HA-LRE1* pMS337 *[CEN4 ARS1 LEU2 HHT1-HHF1]*	This study (Fig. 4g)
rad53-K227A CD-pMET25-3HA-LRE1-H3K4A	CSY0460	*MATa ura3-52 leu2-3,112 trp1 his3 Δ[HHT1-HHF1] Δ[HHT2-HHF2] natNT2-rad53-K227A bar1::HIS3 CD-pMET25-3HA-LRE1* pMS337 *[CEN4 ARS1 LEU2 hht1-H3K4A-HHF1]*	This study (Fig. 4g)
jhd2Δ-H3	CSY0256	*MATa ura3-52 leu2-3,112 trp1 his3 Δ[HHT1-HHF1] Δ[HHT2-HHF2] bar1::HIS3 natNT2-pMET25-3HA-LRE1 jhd2::HPH* pMS337 *[CEN4 ARS1 LEU2 HHT1-HHF1]*	This study (5b-c)
jhd2Δ-H3K4A	CSY0257	*MATa ura3-52 leu2-3,112 trp1 his3 Δ[HHT1-HHF1] Δ[HHT2-HHF2] bar1::HIS3 natNT2-pMET25-3HA-LRE1 jhd2::HPH* pMS337 *[CEN4 ARS1 LEU2 hht1-H3K4A-HHF1]*	This study (5b)

rad53-K227A jhd2Δ-H3	CSY0258	*MATa ura3-52 leu2-3,112 trp1 his3 Δ[HHT1-HHF1] Δ[HHT2-HHF2] kanMX4-rad53-K227A bar1::HIS3 natNT2-pMET25-3HA-LRE1 jhd2::HPH* pMS337 *[CEN4 ARS1 LEU2 HHT1-HHF1]*	This study (5b-f)
rad53-K227A jhd2Δ-H3K4A	CSY0259	*MATa ura3-52 leu2-3,112 trp1 his3 Δ[HHT1-HHF1] Δ[HHT2-HHF2] kanMX4-rad53-K227A bar1::HIS3 natNT2-pMET25-3HA-LRE1 jhd2::HPH* pMS337 *[CEN4 ARS1 LEU2 hht1-H3K4A-HHF1]*	This study (5b, 5d, 5f)
rad53-K227A-H3	CSY0416	*MATa ura3-52 leu2-3,112 trp1 his3 Δ[HHT1-HHF1] Δ[HHT2-HHF2] natNT2-rad53-K227A bar1::HIS3 natNT2-pMET25-3HA-LRE1* pMS337 *[CEN4 ARS1 LEU2 HHT1-HHF1]*	This study (Fig. 6b-c, 7d)
rad53-K227A-H3K4A	CSY0417	*MATa ura3-52 leu2-3,112 trp1 his3 Δ[HHT1-HHF1] Δ[HHT2-HHF2] natNT2-rad53-K227A bar1::HIS3 natNT2-pMET25-3HA-LRE1* pMS337 *[CEN4 ARS1 LEU2 hht1-H3K4A-HHF1]*	This study (Fig. 6b-c, 7d)
RAD53-H3K4R	CSY0021	*MATa ura3-52 leu2-3,112 trp1 his3 Δ[HHT1-HHF1] Δ[HHT2-HHF2]* pMS337 *[CEN4 ARS1 LEU2 hht1-H3K4R-HHF1]*	This study (Fig. 7a)
RAD53 pTEF-CAN1-H3	CSY0545	*MATa ura3-52 leu2-3,112 trp1 his3 Δ[HHT1-HHF1] Δ[HHT2-HHF2] bar1::HIS3 natNT2-pMET25-3HA-LRE1 kanMX4-pTEF-CAN1* pMS337 *[CEN4 ARS1 LEU2 HHT1-HHF1]*	This study (Fig. 7c-f)
RAD53 pTEF-CAN1-H3K4A	CSY0546	*MATa ura3-52 leu2-3,112 trp1 his3 Δ[HHT1-HHF1] Δ[HHT2-HHF2] bar1::HIS3 natNT2-pMET25-3HA-LRE1 kanMX4-pTEF-CAN1* pMS337 *[CEN4 ARS1 LEU2 hht1*-H3K4A-*HHF1]*	This study (Fig. 7d-f)
rad53-K227A pTEF-CAN1-H3	CSY0547	*MATa ura3-52 leu2-3,112 trp1 his3 Δ[HHT1-HHF1] Δ[HHT2-HHF2] natNT2-rad53-K227A bar1::HIS3 natNT2-pMET25-3HA-LRE1 kanMX4-pTEF-CAN1* pMS337 *[CEN4 ARS1 LEU2 HHT1-HHF1]*	This study (Fig. 7c-d)
rad53-K227A pTEF-CAN1-H3K4A	CSY0548	*MATa ura3-52 leu2-3,112 trp1 his3 Δ[HHT1-HHF1] Δ[HHT2-HHF2] natNT2-rad53-K227A bar1::HIS3 natNT2-pMET25-3HA-LRE1 kanMX4-pTEF-CAN1* pMS337 *[CEN4 ARS1 LEU2 hht1-H3K4A-HHF1]*	This study (Fig. 7d-e)
sml1Δ rad53-K227A-H3	CSY0112	*MATa ura3-52 leu2-3,112 trp1 his3 Δ[HHT1-HHF1] Δ[HHT2-HHF2] natNT2-rad53-K227A sml1::HPH pMS337 [CEN4 ARS1 LEU2 HHT1-HHF1]*	This study (Fig. S1b-c)
sml1Δ rad53-K227A-H3K4A	CSY0113	*MATa ura3-52 leu2-3,112 trp1 his3 Δ[HHT1-HHF1] Δ[HHT2-HHF2] natNT2-rad53-K227A sml1::HPH pMS337 [CEN4 ARS1 LEU2 hht1*-H3K4A-*HHF1]*	This study (Fig. S1b-c)
sml1Δ mec1Δ-H3	CSY0463	*MATa ura3-52 leu2-3,112 trp1 his3 Δ[HHT1-HHF1] Δ[HHT2-HHF2] sml1::HPH mec1::kanMX4* pMS337 *[CEN4 ARS1 LEU2 HHT1-HHF1]*	This study (Fig. S1b)
sml1Δ mec1Δ-H3K4A	CSY0464	*MATa ura3-52 leu2-3,112 trp1 his3 Δ[HHT1-HHF1] Δ[HHT2-HHF2] sml1::HPH mec1::kanMX4* pMS337 *[CEN4 ARS1 LEU2 hht1-H3K4A-HHF1]*	This study (Fig. S1b)
sml1Δ mec1Δ rad53-K227A-H3	CSY0465	*MATa ura3-52 leu2-3,112 trp1 his3 Δ[HHT1-HHF1] Δ[HHT2-HHF2] natNT2-rad53-K227A sml1::HPH mec1::kanMX4 pMS337 [CEN4 ARS1 LEU2 HHT1-HHF1]*	This study (Fig. S1b)
sml1Δ mec1Δ rad53-K227A-H3K4A	CSY0466	*MATa ura3-52 leu2-3,112 trp1 his3 Δ[HHT1-HHF1] Δ[HHT2-HHF2] natNT2-rad53-K227A sml1::HPH mec1::kanMX4 pMS337 [CEN4 ARS1 LEU2 hht1*-H3K4A-*HHF1]*	This study (Fig. S1b)
sml1Δ rad53Δ-H3	CSY0116	*MATa ura3-52 leu2-3,112 trp1 his3 Δ[HHT1-HHF1] Δ[HHT2-HHF2] sml1::HPH rad53::HIS5 pMS337 [CEN4 ARS1 LEU2 HHT1-HHF1]*	This study (Fig. S1c)
sml1Δ rad53Δ-H3K4A	CSY0117	*MATa ura3-52 leu2-3,112 trp1 his3 Δ[HHT1-HHF1] Δ[HHT2-HHF2] sml1::HPH rad53::HIS5 pMS337 [CEN4 ARS1 LEU2 hht1*-H3K4A-*HHF1]*	This study (Fig. S1c)

WT	CSY0001	*MATa ade2-1 ura3-1 his3-11,15 trp1-1 leu2-3,112 can1-100*	W303, This study (Fig. S1d, S2b)
set1Δ	CSY0002	*MATa ade2-1 ura3-1 his3-11,15 trp1-1 leu2-3,112 can1-100 set1::kanMX4*	This study (Fig. S1d)
rad53-11	CSY0003	*MATa ade2-1 ura3-1 his3-11,15 trp1-1 leu2-3,112 can1-100 TRP1-rad53-11*	This study (Fig. S1d)
rad53-K227A	CSY0004	*MATa ade2-1 ura3-1 his3-11,15 trp1-1 leu2-3,112 can1-100 natNT2-rad53-K227A*	This study (Fig. S1d, S2b)
rad53-11 set1Δ	CSY0005	*MATa ade2-1 ura3-1 his3-11,15 trp1-1 leu2-3,112 can1-100 TRP1-rad53-11 set1::kanMX4*	This study (Fig. S1d)
rad53-K227A set1Δ	CSY0006	*MATa ade2-1 ura3-1 his3-11,15 trp1-1 leu2-3,112 can1-100 atNT2-rad53-K227A set::kanMX4*	This study (Fig. S1d)
rnh1Δ-H3	CSY0220	*MATa ura3-52 leu2-3,112 trp1 his3 Δ[HHT1-HHF1] Δ[HHT2-HHF2] rnh1::kanMX4* pMS337 *[CEN4 ARS1 LEU2 HHT1-HHF1]*	This study (Fig. S2a)
rnh201Δ-H3	CSY0221	*MATa ura3-52 leu2-3,112 trp1 his3 Δ[HHT1-HHF1] Δ[HHT2-HHF2] rnh201::kanMX4* pMS337 *[CEN4 ARS1 LEU2 HHT1-HHF1]*	This study (Fig. S2a)
rnh1Δ rnh201Δ-H3	CSY0222	*MATa ura3-52 leu2-3,112 trp1 his3 Δ[HHT1-HHF1] Δ[HHT2-HHF2] rnh1::kanMX4 rnh201::HIS5* pMS337 *[CEN4 ARS1 LEU2 HHT1-HHF1]*	This study (Fig. S2a)
rad53-K227A rnh1Δ-H3	CSY0224	*MATa ura3-52 leu2-3,112 trp1 his3 Δ[HHT1-HHF1] Δ[HHT2-HHF2] natNT2-rad53-K227A rnh1::kanMX4 pMS337 [CEN4 ARS1 LEU2 HHT1-HHF1]*	This study (Fig. S2a)
rad53-K227A rnh201Δ-H3	CSY0225	*MATa ura3-52 leu2-3,112 trp1 his3 Δ[HHT1-HHF1] Δ[HHT2-HHF2] natNT2-rad53-K227A rnh201::kanMX4 pMS337 [CEN4 ARS1 LEU2 HHT1-HHF1]*	This study (Fig. S2a)
rad53-K227A rnh1Δ rnh201Δ-H3	CSY0226	*MATa ura3-52 leu2-3,112 trp1 his3 Δ[HHT1-HHF1] Δ[HHT2-HHF2] natNT2-rad53-K227A rnh1::kanMX4 rnh201::HIS5 pMS337 [CEN4 ARS1 LEU2 HHT1-HHF1]*	This study (Fig. S2a)
rnh1Δ-H3K4A	CSY0228	*MATa ura3-52 leu2-3,112 trp1 his3 Δ[HHT1-HHF1] Δ[HHT2-HHF2] rnh1::kanMX4* pMS337 *[CEN4 ARS1 LEU2 hht1-H3K4A-HHF1]*	This study (Fig. S2a)
rnh201Δ-H3K4A	CSY0229	*MATa ura3-52 leu2-3,112 trp1 his3 Δ[HHT1-HHF1] Δ[HHT2-HHF2] rnh201::kanMX4* pMS337 *[CEN4 ARS1 LEU2 hht1-H3K4A-HHF1]*	This study (Fig. S2a)
rnh1Δ rnh201Δ-H3K4A	CSY0230	*MATa ura3-52 leu2-3,112 trp1 his3 Δ[HHT1-HHF1] Δ[HHT2-HHF2] rnh1::kanMX4 rnh201::HIS5* pMS337 *[CEN4 ARS1 LEU2 hht1-H3K4A-HHF1]*	This study (Fig. S2a)
rad53-K227A rnh1Δ-H3K4A	CSY0232	*MATa ura3-52 leu2-3,112 trp1 his3 Δ[HHT1-HHF1] Δ[HHT2-HHF2] natNT2-rad53-K227A rnh1::kanMX4 pMS337 [CEN4 ARS1 LEU2 hht1-H3K4A-HHF1]*	This study (Fig. S2a)
rad53-K227A rnh201Δ-H3K4A	CSY0233	*MATa ura3-52 leu2-3,112 trp1 his3 Δ[HHT1-HHF1] Δ[HHT2-HHF2] natNT2-rad53-K227A rnh201::kanMX4 pMS337 [CEN4 ARS1 LEU2 hht1-H3K4A-HHF1]*	This study (Fig. S2a)
rad53-K227A rnh1Δ rnh201Δ-H3K4A	CSY0234	*MATa ura3-52 leu2-3,112 trp1 his3 Δ[HHT1-HHF1] Δ[HHT2-HHF2] natNT2-rad53-K227A rnh1::kanMX4 rnh201::HIS5 pMS337 [CEN4 ARS1 LEU2 hht1-H3K4A-HHF1]*	This study (Fig. S2a)
mlp1Δ	CSY0572	*MATa ade2-1 ura3-1 his3-11,15 trp1-1 leu2-3,112 can1-100 mlp1::kanMX4*	This study (Fig. S2b)
sac3Δ	CSY0573	*MATa ade2-1 ura3-1 his3-11,15 trp1-1 leu2-3,112 can1-100 sac3::kanMX4*	This study (Fig. S2b)
set1Δ	CSY0049	*MATa ade2-1 ura3-1 his3-11,15 trp1-1 leu2-3,112 can1-100 set1::HIS5*	This study (Fig. S2b)
rad53-K227A set1Δ	CSY0050	*MATa ade2-1 ura3-1 his3-11,15 trp1-1 leu2-3,112 can1-100 natNT2-rad53-K227A set1::HIS5*	This study (Fig. S2b)
rad53-K227A mlp1Δ	CSY0554	*MATa ade2-1 ura3-1 his3-11,15 trp1-1 leu2-3,112 can1-100 natNT2-rad53-K227A mlp1::kanMX4*	This study (Fig. S2b)
rad53-K227A sac1Δ	CSY0555	*MATa ade2-1 ura3-1 his3-11,15 trp1-1 leu2-3,112 can1-100 natNT2-rad53-K227A sac3::kanMX4*	This study (Fig. S2b)

rad53-K227A set1Δ mlp1Δ	CSY0053	*MATa ade2-1 ura3-1 his3-11,15 trp1-1 leu2-3,112 can1-100 natNT2-rad53-K227A set1::HIS5 mlp1::kanMX4*	This study (Fig. S2b)
rad53-K227A set1Δ sac1Δ	CSY0054	*MATa ade2-1 ura3-1 his3-11,15 trp1-1 leu2-3,112 can1-100 natNT2-rad53-K227A set1::HIS5 sac3::kanMX4*	This study (Fig. S2b)
rrm3Δ-H3	CSY0318	*MATa ura3-52 leu2-3,112 trp1 his3 Δ[HHT1-HHF1] Δ[HHT2-HHF2] bar1::HIS3 natNT2-pMET25-3HA-LRE1 rrm3::HPH* pMS337 *[CEN4 ARS1 LEU2 HHT1-HHF1]*	This study (Fig. S2c)
rrm3Δ-H3K4A	CSY0319	*MATa ura3-52 leu2-3,112 trp1 his3 Δ[HHT1-HHF1] Δ[HHT2-HHF2] bar1::HIS3 natNT2-pMET25-3HA-LRE1 rrm3::HPH* pMS337 *[CEN4 ARS1 LEU2 HHT1-HHF1]*	This study (Fig. S2c)
rad53-K227A rrm3Δ-H3	CSY0320	*MATa ura3-52 leu2-3,112 trp1 his3 Δ[HHT1-HHF1] Δ[HHT2-HHF2] kanMX4-rad53-K227A bar1::HIS3 natNT2-pMET25-3HA-LRE1 rrm3::HPH* pMS337 *[CEN4 ARS1 LEU2 HHT1-HHF1]*	This study (Fig. S2c)
rad53-K227A rrm3Δ-H3K4A	CSY0321	*MATa ura3-52 leu2-3,112 trp1 his3 Δ[HHT1-HHF1] Δ[HHT2-HHF2] kanMX4-rad53-K227A bar1::HIS3 natNT2-pMET25-3HA-LRE1 rrm3::HPH* pMS337 *[CEN4 ARS1 LEU2 hht1-H3K4A-HHF1]*	This study (Fig. S2c)

Supplementary Table 2: Primers used in this study

Experiments	Genes/regions	Sequences (5’ - 3’)	Reference/source/note(s)
Yeast construction	*ρMET25-3HA-LRE1*	(F) GAACCCTCGGCAATTATTTCATACTCCTTCTCAGAA TAGGAAAATGCGTACGCTGCAGGTCGAC<br>(R) CAGGATTTGGCTCTGATATTTGCACATGTTGAGTAT GCGTATTCGGCATCGATGAATTCTCTGTCG	To amplify *kanMX4-pMET25-3HA* fragment from pYM-N36[2] for yeast transformation
	lre1:URA3	(F) AACCCTCGGCAATTATTTCATACTCCTTCTCAGAAT AGGAAAATGAGCAGATTGTACTGAGAGTGC<br>(R) CAATGCCGCTAAGGTTACGATTCACCTTTTTATTAC TTATTATTAAGCTCTAATTTGTGAGTTTAG	To amplify *lre1::URA3* cassette from pRS406 for yeast transformation
	HO-pMET25-3HA-LRE1	(F) CTGAACCCTCGGCAATTATTTCATACTCCTTCTCAG AATAGGAAACTTAGCTCTCATTATTTTTTGCTTTTTC<br>(R) CTCCAAGATTACTGAAAAACCTGAA	To amplify marker-free *HO-pMET25-3HA-LRE1* fragment from CSTY0252
	CD-pMET25-3HA-LRE1	(F) CTGAACCCTCGGCAATTATTTCATACTCCTTCTCAG AATAGGAAACGTTAAGGTTACGATTCACCTTTTT<br>(R) TTTATTATTCTACACTTTATTATTAAAACTACATGC AATCAATGCCTTAGCTCTCATTATTTTTTGCTTTTTC	To amplify marker-free *CD-pMET25-3HA-LRE1* fragment from CSTY0252
	pTEF-CAN1	(F) TTCTTAACTCCTGTAAAAACAAAAAAAAAAAAAAGGC ATAGCAATGCGTACGCTGCAGGTCGAC<br>(R) GTACATATGCTTCTCCTCTATGTCGGCGTCTTCTTT TGAATTTGTCATCGATGAATTCTCTGTCG	To amplify *kanMX4-pTEF* fragment from pYM-N18[2] for yeast transformation
	kanMX_pRS	(F) GGATCCCCGGGTTAATTAAGGCGCGCCAGATCTGT TTAGTTCGGTGATGACGGTGAAAACCT<br>(R) TTTTCGACACTGGATGGCGGCGTTAGTATCGAATC GACAGCCTGATTTAACAAAAATTTAACGCGA	Universal primers for amplification of any markers from pRS plasmid series for replacing *kanMX4* cassette by indicated marker in any mutants from BY4741 deletion library
	kanMX4_U2D2	(F) CGTACGCTGCAGGTCGAC<br>(R) ATCGATGAATTCGAGCTCG	Universal primers for amplification of *kanmx4*::Marker fragment from maker-transformed BY4741 mutants
	BAR1	(F) CGGTACACATTTGTTGCATTTATTA<br>(R) GTGATAGTTTAATGGTCAGAATGGG	Gene knockout
	SET1	(F) GGCAAAGAACAAAGAAATACACAGT<br>(R) TGTTCGTGTTTTCAGGCATTT	
	BRE2	(F) GCTAGGAAACTCCTGATCACATTTA<br>(R) TGGCATTTTGAAATGTTATGTAATG	
	SPP1	(F) GGCGATATTCGATAGACTTAACAGA<br>(R) TCTTCTGCAATATCTAAAGTGCTCA	
	JHD2	(F) TCGTGATATAGAAGTAACGCATTGA<br>(R) CGATCGTGAATAAAAATGCTACTCT	
	MEC1	(F).ATTCCTTTTCAAGGCTCCATAACTA<br>(R) TTTTCCATATCTTCGAGCTCTTCTA	
	sml1_hphNT1	(F) GGTCTCACTAACCTCTCTTCAACTGCTCAATAATTT	

		CCCGCTATGCGTAC GCTGCAGGTCGAC<br>(R) GTGGGAAATGGAAAGAGAAAAGAAAAGAGTATGAA GGAACTTTAATCGATGAATTCGAGCTCG	
	rad53_pUG73[3]	(F) AGAATAGTGAGAAAAGATAGTGTTACACAACATCAA CTAAAAATGCAGCTGAAGCTTCGTACGC<br>(R) TCTTAAAAAGGGGCAGCATTTTCTATGGGTATTTGT CCTTGGTTAGCATAGGCCACTAGTGGATCTG	
	RNH1	(F) CATGTATAAAGCACACTGCACCTAC<br>(R) GATGAAAAACTCGAAGAAATTGAAA	
	RNH201	(F) AATTGACAGGAAACAAAACTGAGAC<br>(R) ACAAAGGCAGACATAGTACGCTAAT	
	MLP1	(F) ATCTTTGTTTCCTTTCATTCCTCTT<br>(R) GGAACACTAAGAAAATCTGTTTGGA	
	SAC3	(F) AGAATTCGTGATTCAATGTTCATTAT<br>(R) AGCAGTTTATAACTTTTTGGCCTCT	
	RRM3	(F) TATCTTCCCTTACCGGATTTATTTC<br>(R) GGCTAGATCTCCTTTTTCAGTTTCT	
	rad53-K227A		Integration of *Eco*RI-linearized PCH8-*rad53-K227A* plasmid at the *RAD53* locus[4]
Site-directed mutagenesis	H3R2A	ACGCAAACAATGGCCGCAACAAAGCAAACAGCA	To generate H3 mutant plasmids (pMS337) by PCR mutagenesis[1]. Underlined codons represent mutagenesis in indicated plasmids
	H3K4A	ACAATGGCCAGAACAGCGCAAACAGCAAGAAAG	
	H3K4R	ACAATGGCCAGAACAAGGCAAACAGCAAGAAAG	
	H3K36A	TCTACCGGTGGTGGTTGCGAAGCCTCACAGATAT	
	H3K79A	ATCGCTCAAGATTTCGCGACCGACTTGAGATTT	
Gene expression (qPCR)	*pMET25-3HA-LRE1*	(F) GCGGCTGAAAATTGTCCTT<br>(R) TGTAGCGTATTGGCGATAATGT	This study (Fig. 5d, S4a)
	SCR1	(F) CGGCTAGACACGGATTG<br>(R) GAGGAAAGACGGTTGCCA	This study (Fig. 5d)
	ACT1	(F) TCACGCCATTTTGAGAATCG<br>(R) TTCAGCAGTGGTGGAGAAAGAG	This study (Fig.S4a)
ChIP-qPCR	*pMET25-3HA-LRE1* (P1)	(F) TATTACCCCCATCCATACTCTTGAA<br>(R) ATAGGGATAGCCCGCATAGTCAGGA	This study (Fig. 5e, S4c)
	pMET25-3HA-LRE1 (P2)	(F) TTGAAGACAAGGCCTAGGTCATTT<br>(R) ACAATTGCCCTTATCTACCATAACC	
	pMET25-3HA-LRE1 (P3)	(F) CATTAATGCACAGTCCATGCCCTTT<br>(R) TGCATCGTCCGCTCTCGTAATAAC	
	CAN1 (P1)	(F) ATGACAAATTCAAAAGAAGACGCCG<br>(R) TGAAGCTCCAACGTCGTGAAAGAGG	This study (Fig. 7c)
	CAN1 (P2)	(F) CTGGCGGCATGGATTAGTATTTTTT<br>(R) GACCCAGAACTCGAATTCACCGTAA	
	CAN1 (P3)	(F) CCTAAATTCCTGTCAAGGACCACCA<br>(R) CTCCATGTAAGCCAAAGCGCCAAAT	
	TEL06R (TEL)	(F) CGTGTGTAGTGATCCGAACTCAGT	This study (Fig. 7c, S4c)

		(R) GACCAGTCCTCATTTCCATCAATAG	
2D electrophoresis<br>(DNA fragments for probes)	*ARS305*	(F) AGGTTCAGTATTGAAAGTTGGTA<br>(R) ACTATTTACATTATCGTCCTGTCA	This study (Fig 4c, 4e-f, 5f, 6c)
	ARS305 (HO/CD-*LRE1*)	(F) GCGGCTGAAAATTGTCCTT<br>(R) TTACTTCCTTGAAAAAATATGTCTA	This study (Fig 4g)
	ARS1211 (*PDC1*)	(F) CAAATATCGTTTGAATATTTTTCCG<br>(R) TACACTAATGCAGTTTCAGGGTTTT	This study (Fig 4h)
	ARS305L	(F) CATGAACTTTGTATCTGAATTTTTG<br>(R) ATCTAACTACAAACTGAAGCAGAAT	This study (Fig 6c)
	ARS305LL	(F) GTAATAGCTGCTGATTGGTCAGAAT<br>(R) GAATCTTTCGTCTCGATAAACTTCA	
	ARS1211	(F) CATGGATGTACAGTAGAAATTACAT<br>(R) TGATTTCTATAAATTGGATGTCGAT	
	ARS1211R	(F) TTGAATAGAATCCGTCTCTGCATCA<br>(R) AGGGTGCAAATAACATGTATGAACT	
	ARS1211RR	(F) CTTGTCGATTGTTGTCAGATTACAC<br>(R) TCGTATTGCAATTTTTGTTTGTCTA	
	ARS1212	(F) AATAAGAAGGGAGAGGTGGTAGAGA<br>(R) TATATTGCTGTGATCTGAATCTGGA	

References

1. Recht J*, et al.* Histone chaperone Asf1 is required for histone H3 lysine 56 acetylation, a modification associated with S phase in mitosis and meiosis. *Proc Natl Acad Sci U S A* **103**, 6988-6993 (2006).

2. Janke C*, et al.* A versatile toolbox for PCR-based tagging of yeast genes: new fluorescent proteins, more markers and promoter substitution cassettes. *Yeast* **21**, 947-962 (2004).

3. Gueldener U, Heinisch J, Koehler GJ, Voss D, Hegemann JH. A second set of loxP marker cassettes for Cre-mediated multiple gene knockouts in budding yeast. *Nucleic Acids Res* **30**, (2002).

4. Pellicioli A*, et al.* Activation of Rad53 kinase in response to DNA damage and its effect in modulating phosphorylation of the lagging strand DNA polymerase. *Embo Journal* **18**, 6561-6572 (1999).
