# Supplementary material for: H3K4 methylation at active genes mitigates transcription-replication conflicts during replication stress
Source: Nat Commun. 2020 Feb 10;11:809. doi: 10.1038/s41467-020-14595-4 (PMC7010754; doi:10.1038/s41467-020-14595-4)
Supplement: Supplementary file 3 — Reporting Summary [file 41467_2020_14595_MOESM3_ESM.pdf]

nature research

Corresponding author(s): Yi-Chen Lo and Cheng-Fu Kao
Last updated by author(s): Dec 19, 2019

Reporting Summary

Nature Research wishes to improve the reproducibility of the work that we publish. This form provides structure for consistency and transparency in reporting. For further information on Nature Research policies, see Authors & Referees and the Editorial Policy Checklist.

Statistics

For all statistical analyses, confirm that the following items are present in the figure legend, table legend, main text, or Methods section.

n/a	Confirmed	
☐	☒	The exact sample size (*n*) for each experimental group/condition, given as a discrete number and unit of measurement
☐	☒	A statement on whether measurements were taken from distinct samples or whether the same sample was measured repeatedly
☐	☒	The statistical test(s) used AND whether they are one- or two-sided<br>*Only common tests should be described solely by name; describe more complex techniques in the Methods section.*
☒	☐	A description of all covariates tested
☒	☐	A description of any assumptions or corrections, such as tests of normality and adjustment for multiple comparisons
☒	☐	A full description of the statistical parameters including central tendency (e.g. means) or other basic estimates (e.g. regression coefficient) AND variation (e.g. standard deviation) or associated estimates of uncertainty (e.g. confidence intervals)
☒	☐	For null hypothesis testing, the test statistic (e.g. *F*, *t*, *r*) with confidence intervals, effect sizes, degrees of freedom and *P* value noted<br>*Give P values as exact values whenever suitable.*
☒	☐	For Bayesian analysis, information on the choice of priors and Markov chain Monte Carlo settings
☒	☐	For hierarchical and complex designs, identification of the appropriate level for tests and full reporting of outcomes
☒	☐	Estimates of effect sizes (e.g. Cohen's *d*, Pearson's *r*), indicating how they were calculated

Our web collection on statistics for biologists contains articles on many of the points above.

Software and code

Policy information about availability of computer code

Data collection	*Provide a description of all commercial, open source and custom code used to collect the data in this study, specifying the version used OR state that no software was used.*
Data analysis	Microsoft Excel for basic statistical analysis, PASW Statistic 18 for Mann-Whitney Test.

For manuscripts utilizing custom algorithms or software that are central to the research but not yet described in published literature, software must be made available to editors/reviewers. We strongly encourage code deposition in a community repository (e.g. GitHub). See the Nature Research guidelines for submitting code & software for further information.

Data

Policy information about availability of data

All manuscripts must include a data availability statement. This statement should provide the following information, where applicable:

- Accession codes, unique identifiers, or web links for publicly available datasets
- A list of figures that have associated raw data
- A description of any restrictions on data availability

The ChIP-seq data sets generated during and/or analysed during the current study are available in the GEO repository, [https://www.ncbi.nlm.nih.gov/geo/query/acc.cgi?acc=GSE133222].

Field-specific reporting

Please select the one below that is the best fit for your research. If you are not sure, read the appropriate sections before making your selection.

☒ Life sciences ☐ Behavioural & social sciences ☐ Ecological, evolutionary & environmental sciences

For a reference copy of the document with all sections, see nature.com/documents/nr-reporting-summary-flat.pdf

Life sciences study design

All studies must disclose on these points even when the disclosure is negative.

Sample size	Sample size (n) in mutation assay were determined on the individual colony.
Data exclusions	Some mutation assay data (n<2) from certain groups were excluded due to reverted colonies were too much to count. However, we performed additional experiment to let n=11.
Replication	The finding in this paper were remarkably reproducible. Every important significant phenotype was represented multiple times with the similar outcomes among different experiments: (Fig. 1a, 1c, S1 and S2), (Fig. 3a, 4c and 5b) and (Fig. 4c, 4e-f, 4h and 5f).
Randomization	N/A
Blinding	N/A

Reporting for specific materials, systems and methods

We require information from authors about some types of materials, experimental systems and methods used in many studies. Here, indicate whether each material, system or method listed is relevant to your study. If you are not sure if a list item applies to your research, read the appropriate section before selecting a response.

Materials & experimental systems

n/a	Involved in the study	
☐	☒	Antibodies
☒	☐	Eukaryotic cell lines
☒	☐	Palaeontology
☒	☐	Animals and other organisms
☒	☐	Human research participants
☒	☐	Clinical data

Methods

n/a	Involved in the study	
☐	☒	ChIP-seq
☐	☒	Flow cytometry
☒	☐	MRI-based neuroimaging

Antibodies

Antibodies used

α-H2A S129-P
α-H3K4me3
α-H3K4me2
α-H3K4me1
α-H3
α-HA
α-G6PDH

Validation

α-H2A S129-P (Abcam, ab15083), refs: PubMed: 30654749, 30808869, 29741650...etc (63 publications)
α-H3K4me3 (Abcam, ab8580), refs: PubMed: 30604769, 30616642, 30620009...etc (1247 publications)
α-H3K4me2 (Abcam, ab7766), refs: PubMed: 30648969, 30775443, 30835260...etc (221 publications)
α-H3K4me1 (Abcam, ab8895), refs: PubMed: 30604769, 30631055, 30657937...etc (623 publications)
α-H3 (Abcam, ab1791), refs: PubMed: 30407559, 30599905, 29791073...etc (2745 publications)
α-HA (Roche, 11867423001), validated in our study using western blot assay on HA-tagged protein
α-G6PDH (Sigma, A9521), refs: PubMed: 19139279, 17041589, 26241481...etc (79 publications)

ChIP-seq

Data deposition

☒ Confirm that both raw and final processed data have been deposited in a public database such as GEO.

☒ Confirm that you have deposited or provided access to graph files (e.g. BED files) for the called peaks.

Data access links<br>*May remain private before publication.*	https://www.ncbi.nlm.nih.gov/geo/query/acc.cgi?acc=GSE133222
Files in database submission	GSM3902524 WT_DNAPOL2_input<br>GSM3902525 WT_DNAPOL2_ChIP<br>GSM3902526 H3K4A_DNAPOL2_input<br>GSM3902527 H3K4A_DNAPOL2_ChIP<br>GSM3902528 rad53_DNAPOL2_input<br>GSM3902529 rad53_DNAPOL2_ChIP<br>GSM3902530 rad53_H3K4A_DNAPOL2_input<br>GSM3902531 rad53_H3K4A_DNAPOL2_ChIP<br>GSM3902532 WT_Rpb3_input<br>GSM3902533 WT_Rpb3_ChIP<br>GSM3902534 H3K4A_Rpb3_input<br>GSM3902535 H3K4A_Rpb3_ChIP<br>GSM3902536 rad53_Rpb3_input<br>GSM3902537 rad53_Rpb3_ChIP<br>GSM3902538 rad53_H3K4A_Rpb3_input<br>GSM3902539 rad53_H3K4A_Rpb3_ChIP
Genome browser session<br>(e.g. UCSC)	https://www.ncbi.nlm.nih.gov/geo/query/acc.cgi?acc=GSE133222

Methodology

Replicates	One replicate
Sequencing depth	*Describe the sequencing depth for each experiment, providing the total number of reads, uniquely mapped reads, length of reads and whether they were paired- or single-end.*
Antibodies	α-Rpb3 (Biolegend, 665003)<br>α-FLAG (Sigma, F3165)
Peak calling parameters	*Specify the command line program and parameters used for read mapping and peak calling, including the ChIP, control and index files used.*
Data quality	*Describe the methods used to ensure data quality in full detail, including how many peaks are at FDR 5% and above 5-fold enrichment.*
Software	FASTQ files from biological and technical replicates for each sample were merged. Reads were aligned to the sacCer3 reference genome (release R64-2-1) using Bowtie2 version 2.2.5 in 'very-sensitive' mode. Aligned reads were filtered and indexed using SAMtools. Reads were adjusted so that the genome average was set at 1-fold enrichment, then data from immunoprecipitated samples were divided by data from input samples. Peak calling was completed using the "callpeak" command in MACS v2 software, and shared peaks between samples were determined using the MACS "bdgdiff" command. Heatmaps were generated using deepTools. Transcription frequency was calculated by a sum of signal in a range TSS to TSS + 200 bp of each yeast gene adapted from the results of NET-seq.

Flow Cytometry

Plots

Confirm that:

☒ The axis labels state the marker and fluorochrome used (e.g. CD4-FITC).

☒ The axis scales are clearly visible. Include numbers along axes only for bottom left plot of group (a 'group' is an analysis of identical markers).

☒ All plots are contour plots with outliers or pseudocolor plots.

☒ A numerical value for number of cells or percentage (with statistics) is provided.

Methodology

Sample preparation	Cells were fixed in 70% ethanol and stained DNA with SYBR Green.
Instrument	BD FACSCanto™ II
Software	FACS DIVA software (BD Biosciences) and FlowJo V7.6.1
Cell population abundance	10,000 gated cells for DNA content measurement

Gating strategy

1n and 2n DNA signal based on log-phase cell sample of each group.

☐ Tick this box to confirm that a figure exemplifying the gating strategy is provided in the Supplementary Information.
